# Principles for task shifting hypertension and diabetes screening and referral: a qualitative study exploring patient, community health worker and healthcare professional perceptions in rural Uganda

**DOI:** 10.1186/s12889-023-15704-w

**Published:** 2023-05-12

**Authors:** Rebecca Ingenhoff, Richard Munana, Ivan Weswa, Julia Gaal, Isaac Sekitoleko, Hillary Mutabazi, Benjamin E. Bodnar, Tracy L. Rabin, Trishul Siddharthan, Robert Kalyesubula, Felix Knauf, Christine K. Nalwadda

**Affiliations:** 1 Department of Nephrology and Medical Intensive Care, Charité - Universitätsmedizin Berlin, Berlin, Germany; 2 Department of Community Health and Behavioural Sciences, School of Public Health, Makerere University College of Health Sciences, Makerere University, Kampala, Uganda; 3 African Community Center for Social Sustainability, Nakaseke, Uganda; 4 MRC/UVRI and LSHTM Uganda Research Unit, Kampala, Uganda; 5 London School of Hygiene and Tropical Medicine, London, UK; 6 Department of Medicine, Johns Hopkins University School of Medicine, Baltimore, MD USA; 7 Department of Internal Medicine, Yale School of Medicine, New Haven, CT USA; 8 Department of Pulmonary, Critical Care and Sleep Medicine, University of Miami, Coral Gables, USA; 9 Department of Physiology, Department of Internal Medicine, Makerere University College of Health Sciences, Kampala, Uganda

**Keywords:** Community health workers, Perceptions, Task shifting, Screening, Referral, Hypertension, Diabetes, Uganda

## Abstract

**Background:**

A shortage of healthcare workers in low- and middle-income countries (LMICs) combined with a rising burden of non-communicable diseases (NCDs) like hypertension and diabetes mellitus has resulted in increasing gaps in care delivery for NCDs. As community health workers (CHWs) often play an established role in LMIC healthcare systems, these programs could be leveraged to strengthen healthcare access. The objective of this study was to explore perceptions of task shifting screening and referral for hypertension and diabetes to CHWs in rural Uganda.

**Methods:**

This qualitative, exploratory study was conducted in August 2021 among patients, CHWs and healthcare professionals. Through 24 in-depth interviews and ten focus group discussions, we investigated perceptions of task shifting to CHWs in the screening and referral of NCDs in Nakaseke, rural Uganda. This study employed a holistic approach targeting stakeholders involved in the implementation of task shifting programs. All interviews were audio-recorded, transcribed verbatim, and analyzed thematically guided by the framework method.

**Results:**

Analysis identified elements likely to be required for successful program implementation in this context. Fundamental drivers of CHW programs included structured supervision, patients’ access to care through CHWs, community involvement, remuneration and facilitation, as well as building CHW knowledge and skills through training. Additional enablers comprised specific CHW characteristics such as confidence, commitment and motivation, as well as social relations and empathy. Lastly, socioemotional aspects such as trust, virtuous behavior, recognition in the community, and the presence of mutual respect were reported to be critical to the success of task shifting programs.

**Conclusion:**

CHWs are perceived as a useful resource when task shifting NCD screening and referral for hypertension and diabetes from facility-based healthcare workers. Before implementation of a task shifting program, it is essential to consider the multiple layers of needs portrayed in this study. This ensures a successful program that overcomes community concerns and may serve as guidance to implement task shifting in similar settings.

**Supplementary Information:**

The online version contains supplementary material available at 10.1186/s12889-023-15704-w.

## Introduction

The Alma Ata Declaration on primary health care, established health as a human right while highlighting the significance of community health workers (CHWs) [1]. Over 40 years later, improving the access to healthcare by bridging the care gap between communities and health centers remains a major challenge for low- and middle-income countries (LMICs) like Uganda. Uganda is experiencing a shortage of health care workers, particularly in rural areas [2]. Simultaneously, the global rise of non-communicable diseases (NCDs) continues unabated. In Uganda, this dual burden puts a strain on the already fragile healthcare system [2]. On a global scale, NCDs such as hypertension and diabetes mellitus (diabetes) account for over 70% of yearly deaths worldwide. This is an alarming trend particularly in LMICs, where 80% of these deaths occur [3].

Currently 1.13 billion people suffer from hypertension worldwide, and the majority of these individuals live in LMICs [4]. Diabetes, a major driver of global mortality and morbidity, currently affects 8.5% of adults, with an increase in cases documented over the past two decades [3, 5]. The WHO estimates that 1.5 million deaths were directly caused by diabetes in 2019. Diabetes and hypertension are both major risk factors for heart attacks and strokes, and multi-morbidity is unfortunately common [5]. Uganda is severely burdened by NCDs, which account for more than 40% of yearly deaths [6]. Current numbers indicate that 26.4% of Ugandans suffer from hypertension while 1.4% of the Ugandan population are estimated to have diabetes [7, 8]. The majority of NCDs in Uganda remain underdiagnosed, generating a prevention and care gap that may be closed through the implementation of community-based disease management programs [8–11].

To overcome these challenges in workforce and access to healthcare, the Uganda Ministry of Health initiated the CHW program called the Village Health Teams (VHTs). VHTs typically are volunteers who provide a connection between the health sector and the communities in which they live [12, 13]. In the study region, CHWs are nominated by their respective communities often with at least seven years of education, along with proficiency in reading and writing in Luganda, the community’s most commonly used local language. The Ugandan Ministry of Health provides foundational health training to CHWs prior to serving in local communities, where they may aid in data collection, health education matters or disease surveillance. Despite working as volunteers, they often receive a small contribution to cover their travel expenses based on the activity conducted [12]. While CHWs are well utilized in the management of communicable diseases such as malaria, tuberculosis and HIV/AIDS, evidence on employing CHWs in the management of chronic diseases is limited [14, 15].

Task shifting, where tasks are delegated from physicians to nurses or lay health workers, has been successfully implemented and proven to enhance access, coverage and quality of care [15, 16]. Task shifting to non-physician health workers may be a feasible method to tackle the NCD burden in LMICs, where a shortage of health workers is experienced [15, 17, 18]. With the alarming rise of NCDs in Uganda, efforts are being made to scale-up task shifting to CHWs programs [12, 13]. In 2018, the Ministry of Health in Uganda introduced the nation-wide Community Health Extension Workers program to institutionalize the CHW role and ensure a balanced division of CHWs across the country [19]. However, challenges of acceptance and programmatic components persist [2, 12, 14, 20]. CHWs require professional management and supervision, available equipment and supplies, as well as continuous training. Recent evidence from rural Uganda suggests that the program currently faces several challenges related to CHWs’ job motivation, satisfaction and inherently their performance. Additionally, CHWs often face difficulties with transportation due to a lack of financial resources, as well as a lack of recognition from higher levels of the healthcare system [21].

Reliable evidence is lacking regarding the design of task shifting to CHWs NCD programs, particularly in rural areas. Integrated multi-stakeholder studies investigating the acceptance of community-based NCD programs are scarce [20]. The purpose of this study was to explore patient, CHW and healthcare professional (HCPs) perceptions of a task shifting to CHWs intervention for the screening and referral of hypertension and diabetes in Nakaseke, rural Uganda before the introduction of such a program. During the planned intervention, trained CHWs perform hypertension and diabetes screening using blood pressure devices and glucometers at the household level. CHWs then direct patients matching the referral criteria to the nearest health center. Input from all stakeholders was expected to be vital to inform a holistic understanding of the complex care environment.

## Methods

### Study design

We carried out an exploratory, qualitative study using focus group discussions (FGDs) with patients and in-depth interviews (IDIs) with CHWs and HCPs. Participants were selected through a purposeful sampling approach to represent similar characteristics such as disease status or occupation in the three participant groups. A heterogeneous sample of patients featuring diversity in age, gender and disease status was selected from a prior community census [22]. This qualitative study is one component of a larger mixed-methods investigation on the impact of CHW-led community-based screening and referral for hypertension and diabetes in rural Uganda [23].

### Study site

Nakaseke is a rural district in Central Uganda approximately 66 km north of Kampala with an estimated population of 202,200 [24]. It consists of nine sub-counties including Semuto Town Council and Nakaseke Town Council. Nakaseke district is home to approximately 43,000 households, with a gender composition of 53.1% males and 46.9% females. There is a high rate of illiteracy, with 27% of individuals aged 18 years and above reported to be illiterate [25]. The study site was selected purposively due to a prior community census and the research infrastructure available [22]. The population profile of the study population is representative of rural populations in Uganda, yet the pre-established cohort presents a unique characteristic for the study’s design.

### Participants

Patients who currently lived in Nakaseke district were recruited from two public hospitals in the district. CHWs were inhabitants of Nakaseke district and attached to a local non-governmental organization (NGO) the African Community Center for Social Sustainability Uganda (ACCESS). The HCPs were either from or worked in Nakaseke district local government, Nakaseke Hospital or in the capital city Kampala with expertise in national NCD or CHW programming.

### Data collection

The study was conducted in Nakaseke district, rural Uganda in August 2021. A small number of HCPs’ interviews were conducted in Kampala, Uganda. Three distinct, interview guides were developed. Interview guide 1 was utilized for the patient FGDs, focusing on personal experiences and perceptions of CHWs. Interview guide 2 was designed for the IDIs with CHWs, focusing on their needs and experiences. Interview guide 3 was used for HCPs, encompassing structural and professional experiences with CHWs. Interview guides were initially written in English and translated to Luganda by the Makerere University Multilingual Project to ensure validity. Data was collected by research assistants who were social scientists, fluent both in English and Luganda, the most commonly used dialect in the study area. The research assistants were trained to understand the study objectives, data collection tools and supervised by two project managers from ACCESS Uganda with prior experience in managing community-based research studies. Pilot interviews and pilot FGDs were conducted by the local project managers and research assistants after which the data collection tools were redesigned to fit the research needs and local context. Participants were either contacted by phone or in-person. Patient interviews were conducted in Luganda, HCP interviews were conducted in English, and CHW interviews were held in English or Luganda, based on participant preference. Interviews in Luganda were recorded and transcribed verbatim to English by research assistants. Interviews obtained in English were recorded and transcribed in English. The location for conducting the majority of patient FGDs was outdoors on the grounds of Nakaseke District Hospital or Semuto Health Centre IV, one FGD was performed within the community. IDIs with CHWs were conducted at ACCESS Uganda. The HCPs’ IDIs were carried out at the participants’ respective place of work such as the Ministry of Health, Nakaseke District Hospital, Semuto Health Centre IV, and ACCESS Uganda. Due to the ongoing COVID-19 pandemic at the time of data collection and inherent movement restrictions, some interviews were facilitated digitally via video software. IDIs typically lasted between 30 and 45 min, FGDs took approximately one hour.

### Analysis

We used an inductive approach to thematic analysis where the identified themes are strongly linked to the data [26, 27]. By re-reading transcripts individually to identify reoccurring topics and discrepancies, RI and RM collaborated to create the initial code book using independent line-by-line coding of a sample of transcripts with Microsoft Word. As the transcripts offer a large dataset, codes were used repeatedly. RI and RM agreed on a set of codes that were arranged in categories to capture emerging themes. At the next stage, the initial codebook was discussed and further refined collaboratively by RI, RM and CN, as well as based on the feedback from the research team. After agreeing on a final thematic framework, RI applied the codes to the remaining transcripts using MAXQDA qualitative analysis software. Data was analyzed using the framework method as a proven concept in multi-disciplinary health research, allowing for a thematic or qualitative content approach [28].

### Ethical approval and consent

Ethical approval for this study was granted by the Makerere University School of Biomedical Sciences (SBS-REC 874) and the Uganda National Council of Science and Technology (SS821ES). All participants were compensated for their time and effort, provided written informed consent to participate in the study and agreed to their interview data being published anonymized. The study was conducted in line with the required Covid-19 regulations such as wearing personal protective equipment, sanitizing and social distancing.

## Results

A total of 101 participants participated in the research, grouped into three categories: HCPs, CHWs and patients (Table 1). We conducted individual IDIs with twelve CHWs and twelve HCPs. Further, we conducted ten FGDs with 77 patients in total. Patients who were previously diagnosed with either hypertension, diabetes or both, and were currently seeking care at either Nakaseke District Hospital or Semuto Health Centre IV were selected purposeful from a previous census study on NCDs in Nakaseke [22]. The local research team identified these patients from clinical and research records. Patients that were willing to share their perceptions of the study’s topic and available to participate in the FGD at the respective study site were selected. Individual FGDs were created while ensuring a diversity and balance among the demographic factors such as gender and age that were selected in our initial sampling strategy. CHWs working at ACCESS Uganda that runs health and education programs in the local community participated in this study. The local research team contacted CHWs either in person or via telephone to find those who were interested in participating. Only CHWs that previously received basic training on NCDs as part of a prior community census, but were not yet employed in task shifting, were selected for the interviews [22]. HCPs were selected nurses, doctors, and project implementers from ACCESS Uganda, as well as individuals from local, regional and national authorities such as the District Health Office or the Uganda Ministry of Health. HCPs were identified by the local research team due to their reputable knowledge in the field of NCDs and CHW-led disease management programs. We ensured gender balance among the CHW and HCP participants.

**Table 1:** Characteristics of patients, CHWs and HCP that participated in the study

**Characteristics**	**Patients (N = 77)**	CHWs (N = 12)	HCPs (N = 12)
**Age group**			
18–30	7 (9,1%)	5 (41,7%)	0
31–40	6 (7,8%)	2 (16,7%)	4 (33,3%)
41–50	19 (24,7%)	5 (41,7%)	4 (33,3%)
51–60	22 (28,5%)	0	4 (33,3%)
61–70	13 (16,9%)	0	0
71–80	4 (5,2%)	0	0
Age unknown	6 (7,8%)	0	0
**Sex**			
Female	54 (70,1%)	5 (50%)	6 (50%)
Male	23 (29,9%)	5 (50%)	6 (50%)
**Diagnosis**			
Hypertension	15 (19,5%)		
Diabetes mellitus	18 (23,4%)		
Hypertension and Diabetes mellitus	30 (38,9%)		
Hypertension and/or Diabetes mellitus	14 (28,2%)		
**HCP Role**			
Nurse			4 (33,3%)
Doctor			1 (8,3%)
Project Implementer			4 (33,3%)
Government Employee			3 (25%)

**Fig. 1:**
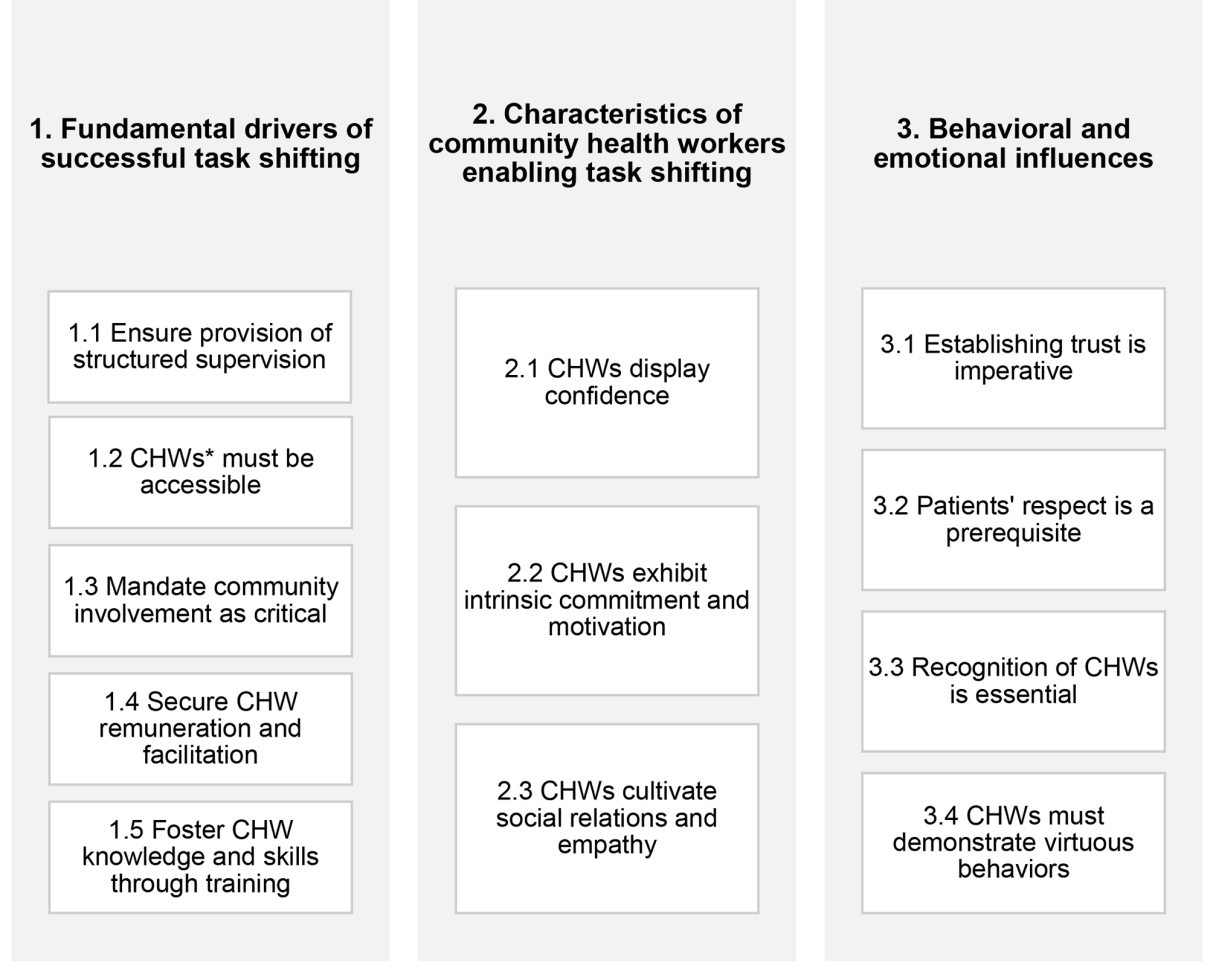
Categories and themes as emerged from the qualitative data ^*^ *Community health workers*

Figure 1 provides an overview of the three categories and twelve themes that emerged from the thematic analysis of interviews regarding patient, CHW and HCP perceptions of task shifting for screening and referral. The categories and themes are presented here, along with representative quotes from each respondent group.

### Fundamental drivers of successful task shifting

As demonstrated in the following quotations, there are a common set of fundamental drivers that all three respondent groups agreed would be important for a successful task shifting implementation.

#### Ensure provision of structured supervision

Participants agreed that a clear supervision structure needs to be in place to communicate challenges and successes. As HCPs argue, CHWs should have clear targets that are monitored while roles are clearly defined and documented. Patients emphasized that supervision is key in successful task shifting.

*‘At community level, we also come to monitor and supervise and see that [CHWs] (…) are actually working within their defined roles.’ (HCP-12)*

*‘CHWs can ably do the screening, identification and referral of diabetic patients if there is close supervision and monitoring (…).’ (Patient, FGD-10)*

Patients laid out that supervision should also include random supervision visits in the community. Moreover, following up with screened patients to leverage their experiences and recommendations towards improving the program.

*‘I would arrange some field visits whereby spot checks will done; interaction with patients who have received CHWs’ services as an evaluation exercise (…) from the community outlook.’ (Patient, FGD-05)*

Regular performance reviews ensure CHWs adhere to guidelines. CHWs perceived supervision and feedback on screening targets as a mode of motivation. Field visits by the supervisors were displayed as positive by CHWs.

*‘Telling us how everyone has performed or how many everyone has [referred] could (…) give us more encouragement and more morale (…).’ (CHW-02)*

#### CHWs must be accessible

This theme was dominated by patients’ voices. CHWs can provide care at the household level, making disease monitoring more accessible and flexible in remote villages. As a patient demonstrated, this can save patients tremendous transport costs.

*‘Our villages are very far away from health facilities, so having a CHW (…) will be extremely important because she will check on me and advise me to go to the health center earlier (…).’ (Patient, FGD-08)*

*‘Transport is very hectic. So having got someone in our community who can do the job, it will save us the more [money].’ (Patient, FGD-05)*

Nonetheless, this theme also featured negative aspects on accessibility as patients had high expectations towards the availability and reliability of CHWs. Patients raised the concern that they need to be able to follow their own work schedules and often find CHWs unavailable when services are needed.

*‘They are not reliable in terms of their availability at their homes. In most cases they are occupied with other kinds of work.’ (Patient, FGD-10)*

To overcome this challenge, a patient suggested that having a dedicated workplace that is separate from the CHWs’ homes would also make it easier for the CHW to separate family and work and formalize the CHWs’ work.

*‘I [would] establish a special place where this CHW will be found within the community. I [would] not put this place at his / her home because this responsibility will interfere [with] her family affairs and privacy. This person [would] have defined days to do home visits and days to be at his / her office.’ (Patient, FGD-08)*

#### Mandate community involvement as critical

The involvement of local leaders was identified as fundamental in task shifting. Local leaders are individuals who offer leadership to their respective communities. They are chosen from among their peers to represent the needs and interests of the community they serve. Local leaders can support CHWs in the program implementation by introducing them to the community. Introductions to the community leader before undertaking activities within a community builds trust between CHWs and community members and nourishes long-term program success. Moreover, local leaders can support CHWs in identifying remote patients.

*‘Community leaders (…) are influential people, (…) including the religious leaders [and] cultural leaders (…). Someone might be in community without a title but (…) when he says something (…), people will follow him.’ (HCP-02)*

Furthermore, patients appear to trust the local leaders in the selection of CHWs but also in the selection of community programs. As a patient described, community meetings provide a platform for discussing challenges.

*‘There should be a village meeting about some of the complaints we have made. (…) On a Sunday, we seat with [the local chairman] in presence of the CHWs.’ (Patient, FGD-01)*

#### Secure CHW remuneration and facilitation

HCPs argued that NCD screening is complex, therefore CHWs need to be formally compensated. An allowance can motivate CHWs in performing their roles, even when they act as volunteers. The suggested amount was between USD 25 and USD 100 per month.

*‘They need to be paid (…). [It] will influence someone to concentrate on this assignment other than giving it divided attention when looking for additional money to meet their financial demands.’ (HCP-04)*

*‘Their work is very complicated because it is not easy to move door to door (…); they deserve to get some allowance.’ (HCP-08)*

A patient demonstrated that CHWs need to support their own lives and families, while expected to prioritize their work and to present a certain level of NCD knowledge. This expected level of education in handling NCDs should be compensated adequately.

*‘There should [be] a motivation (…) so that [CHWs] don’t just work for free (…) So that they know that even if the shoe they are using to walk gets old, they can repair it, if the cloth gets dirty they can be able to buy some soap.’ (Patient, FGD-09)*

Additionally, CHWs need to be given the required equipment to screen for NCDs. Patients acknowledged that other expenses such as transport and phone communication should also be covered to ensure they can fulfill their roles.

*I would want them to get the [screening] machines. (…) To see [my] status on diabetes and hypertension.’ (Patient, FGD-03)*

*‘This person [CHW] will need (…) access to easy transport means. Secondly; (…) a mobile phone for communication. Thirdly; (…) some monthly allowance that will motivate him / her to work.’ (Patient, FGD-08)*

Working while not being able to fulfill basic needs such as eating due to financial constraints limits CHWs in performing their roles.

*‘Being in field and you’re worried on what to eat, we can’t deliver to our maximum.’ (CHW-05)*

Furthermore, CHWs described that at times the community expects them to support vulnerable patients by enabling transport to the nearest hospital, which reinforces the need for securing adequate facilitation and remuneration.

*‘There is need to add on my transport that will enable me to transport some patients found in poor living conditions. Some patients especially the old ones find a challenge of moving (…) to health centers for medication.’(CHW-04)*

#### Foster CHW knowledge and skills through training

Participants agreed that CHWs need to be intensively trained prior to task shifting assignment. Training was regarded as essential for ensuring adherence to medical standards. As a HCP emphasized, continuous training was identified as crucial, including to ensure that CHWs know how to properly use equipment in the field.

*‘They need to be trained well enough (…). Also give them [CHWs] refresher courses wherever gaps are identified.’ (HCP-12)*

In addition, CHWs and HCPs emphasized the need to strengthen patient communication and lifestyle counseling skills so CHWs can approach community members comfortably.

*‘[Train] how to approach someone because if the [CHW] does not know how to talk well with people, [some] may not allow [the CHWs] to talk to them.’ (HCP-03)*

Patients laid out that providing communication skills as part of training is important, even before knowing how to operate the equipment.

*‘I [would] train CHWs on how best to handle NCD patients. I [would] tell them to receive patients with happiness. Then I [would] train them on how to use those machines.’ (Patient, FGD-02)*

Patients’ negative views on CHWs’ current knowledge and skills augmented the requirement for training as they presented doubts whether CHWs have the necessary skills to screen for NCDs.

*‘They [CHWs] had no knowledge on NCDs and they couldn’t help NCD patients (…). These CHWs lack the required knowledge and skills to do this work.’ (Patient, FGD-02)*

### Characteristics of community health workers enabling task shifting

This category was heavily influenced by CHW responses and supported by patients and HCPs, while the perspectives on confidence and intrinsic commitment and motivation were exclusively shaped by the responses of CHWs.

#### CHWs display confidence

CHWs portrayed a high level of confidence towards their abilities on patient communication and performing the outlined tasks of screening and referring hypertensive and diabetic patients. Being trained and appreciated by the community further underlines their confidence in performing their roles.

*‘Being with patients helped boost my confidence.’ (CHW-02)*

*‘I told you about the training I received, I am qualified I understand things and if it’s time to screen a diabetes patient there is nothing to stop me.’ (CHW-06)*

Beyond screening for diseases, CHWs acknowledged their good communication skills. One CHW stated they have the capacity to create a linkage between the patient and the health centers.

*‘We are informed. (…) we are like a linkage between patients and health workers.’ (CHW-12)*

*‘Through the training, (…) I have acquired good communication skills, which help me to convince the patients to seek for health attention.’ (CHW-09)*

#### CHWs exhibit intrinsic commitment and motivation

CHWs demonstrated a high degree of motivation, some even working unpaid. They thrive to empower patients to know their disease status.

*‘I am a CHW and all work is in the community. Personally, I feel motivated to help other people to live a healthy lifestyle.’ (CHW-05)*

The acquired knowledge from training further motivates CHWs to perform their work while being committed to helping patients.

*‘The salary and knowledge they give me, [I] am satisfied because brain [knowledge] is power.’ (CHW-06)*

The role of CHWs makes them feel needed by the community and provides a sense of self-worth, a position where their voices are heard. Also meeting new community members motivates CHWs to perform in their roles. They aim to be a role model for communities.

*‘I make sure that I happen to be the first example before telling others what to do.’ (CHW-09)*

CHWs perceived it as an honor to act as a health worker. The changes on people’s health is a major motivation.

*‘I am happy that there are many people who received our services because of this program and without it; I think they would have been left out.’ (CHW-07)*

In addition, the program influences CHWs and their own families to change their lifestyle habits and to act as a multiplier through the community.

*‘I have become a doctor of my life. I have changed my healthy lifestyle. I cannot sleep without a mosquito net; I cannot treat my child without examination (…).’ (CHW-07)*

#### CHWs cultivate social relations and empathy

Being born in the areas they serve, CHWs are known members of the community. If CHWs are assigned to an area and the community is familiar with them, it will simplify entering the community and communication as a HCP describes.

*‘CHWs are in the communities. They live with the subjects; they will understand them better (…).’ (HCP-12)*

Patients perceived CHWs as equals, following the same occupation as community members while showing commitment and empathy towards their work.

*‘They are not bad, they are like us, because they are farmers like us and you can’t differentiate them from us.’ (Patient, FGD-04)*

*‘CHWs are so transparent and interested in the kind of work they do. This is because they show us love whenever we seek for their health advice (…). I think they behave this way because they come from our communities.’ (Patient, FGD-06)*

HCPs and patients argued that CHWs need to be empathetic when for instance delivering bad news to not create worries among patients.

*‘We need someone who is empathetic towards people’s health.’ (HCP-04)*

Several patients suggested that a CHW should also be a NCD patient as empathy towards the patients would be stronger.

*‘There is need to recruit a CHW who is a patient of NCD. This person will greatly support us because he will know the magnitude of NCDs that his friends are fighting with.’ (Patient, FGD-02)*

### Behavioral aspects and emotional influences

The following results were firmly shaped by patients, who raised needs and concerns that were partly supported by CHWs or HCPs responses.

#### Establishing trust is imperative

HCPs emphasized the need to engage local leaders to build trust. A CHW supported this notion by sharing her personal experience as a counsellor.

*‘We engage local leaders and CHWs to recommend teams of people they trust.’ (HCP-04)*

*‘For the village where I come from, people appreciate me (…). Me, [I] am trusted because [I] am now the woman counsellor where I come from.’ (CHW-06)*

CHWs and patients agreed that carrying medical equipment helps CHWs in building trustful relationships with patients.

*‘Our gadgets and equipment created confidence in community members to accept the exercise.’ (CHW-07)*

*‘If the CHW is well equipped, I will send my children to call him (…).’ (Patient, FGD-02)*

In addition, CHWs laid out that being affiliated to an institution and receiving the support of medical doctors will nurture trust.

*‘We need to have effective communication with medical doctors to give referred patients special attention and in the process, our patients will have confidence in us as community health workers.’ (CHW-11)*

In contrast, patients raised specific concerns about situations that may inhibit trust and the willingness to participate in the program, which underlines that establishing trust is essential. Patients presented doubts about the capability or qualification of CHWs in handling medical devices and the mode of supervision.

*‘Those [screening] machines (…); she [the CHW] may not know how to convert and know if it is (…) in normal range. I will run away (…) because [the CHWS] don’t know what they are doing.’ (Patient, FGD-03)*

A negative experience or personal disagreement with CHWs may lose patients’ trust and impact their health seeking behavior. To support this discussion, some patients even stated that they “despise” people from the community.

*‘If I have personal conflict with that CHW, I cannot go there; I would rather die.’ (Patient, FGD-05)*

Patients presented concerns that CHWs need to keep the health status of patients confidential and not expose it to the community. In addition, patients perceived CHWs’ corrupted behaviors as challenging.

*‘I cannot allow a CHW to measure my blood sugar if s/he is not ready to keep my results confidential. (…) S/he will expose my diabetic results [to] the entire village (…). But if the CHW is good at keeping secrets; then I will be ready to participate (…).’ (Patient, FGD-10)*

*‘It is very bad to hear that some CHW ask for money from patients.’ (Patient, FGD-10)*

#### Patients’ respect is a prerequisite

Respect was described as a major driver of a programs’ success.

‘It takes a lot of understanding (…). That can only be extended to the community members by the people who live with them; whom they believe in; who actually they respect. So that is the vote for CHWs.’ (HCP-12)

Yet, (young) age was identified as a main barrier to respect by HCPs and CHWs, posing a potential challenge to program implementation.

*‘And age, some look to be too young that some people may despise them.’ (HCP-03)*

*‘I think this [lack of respect] results from the mere fact that some of them are older than us, big, tall as opposed to us who are small bodied.’ (CHW-01)*

Underlining this argument, a patient presents that being familiar with a CHW and their upbringing as a barrier to respect. Similarly, a patient stated that knowing a CHWs main occupation such as farming reduces respect.

*‘It is very hard for people to respect someone who comes from within.’ (Patient, FGD-02)*

*‘Even when he brings out a certificate that I am from training, you who was there digging beans [farming]? I cannot.’ (Patient, FGD-04)*

To address this challenge, patients recommended to assign CHWs to villages where they were not brought up.

*‘Now during fieldwork, I will use CHWs of village A to work in village B and vice versa. This will help all teams to be respected in those areas because they will be new to them.’ (Patient, FGD-02)*

#### Recognition of CHWs is essential

HCPs reported that being recognized can drive task shifting success through motivation. Being known in and appreciated by the community empowers CHWs and influences their sense of belonging.

*‘[Providing] a sense of belonging and empowerment in the community. (…) They become [a] point of reference in the community (…) that come[s] with prestige.’ (HCP-10)*

A CHW describes that the recognition from the community increased the responsibility towards their work.

*‘I became responsible with this job and got honor in this job because people call me a health worker (…).’ (CHW-10)*

#### CHWs must demonstrate virtuous behaviors

For successful task shifting, patients stated that CHWs need to be proactive, honest and act as role models. Portraying acceptable social behaviors for instance towards alcohol consumption was identified as important by patients.

*‘Emphasizing them to portray good behaviors within their communities by avoiding getting involved in bad activities like overdrinking which will influence them to misbehave.’ (Patient, FGD-10)*

To further highlight the need for virtuous behaviors, participants described potential misbehaviors of and negative experiences with CHWs that may inhibit the success of a task shifting program. One patient described that a CHW took advantage of a vulnerable situation by asking for physical favors.

*‘Vibing [Pursuing a romantic relationship with] patients, (…) they ask for love and say if you do like this and that, it will be easy for you. Have you gotten it?’ (Patient, FGD-09)*

Underscoring this narrative, patients emphasized that virtuosity encompasses that CHWs focus on providing care rather than prioritizing their income.

*‘In some communities, people lost hope in their CHWs because they are negligent and they are unsupportive.’ (Patient, FGD-06)*

*‘There are [CHWs] who (…) came to make money not to care about the sick.’ (Patient, FGD-01)*

In line to this, patients criticized nepotism and misbehaviors, especially in the dispersion of medication, which as patients argue, CHWs give at times first to relatives and friends.

*‘There is need to select a well behaved and honest person who will not steal the public medicine.’ (Patient, FGD-08)*

## Discussion

The study investigated the principles for introducing task shifting to CHWs. We thereby focused on task shifting screening and referral for hypertension and diabetes in rural Uganda, exploring the perceptions of patients, CHWs and HCPs. Based on these findings, we argue that the success of a task shifting program builds on a set fundamental programmatic structures, while certain CHW characteristics as well as behavioral and socioemotional influences are not to be neglected. Hence, moving beyond paying attention to only programmatic structures to a holistic approach that considers CHW characteristics as well as patients’ concerns through the construction of longitudinal relationships is critical.

Firstly, fundamental underpinnings as outlined and confirmed by all participant groups surround training, supervision, remuneration, community involvement and ease communities’ access to care through CHWs. Roles and tasks need to be clearly defined and monitored by an institution or health center as the World Health Organization guideline on health policy and system support to optimize CHW programs underlines [29]. These programmatic themes were investigated in previous studies in a diverse set of contexts. They were presented as vital to ensure continuity and adherence to standards while including training on patient communication and proper referral, which is consistent with a study performed in the United States [30]. Our results match previous findings that indicate, anchoring the programs in the community by working with trusted leaders will enable community trust and limit patient concerns as access to care advances [11, 29, 31]. Transport facilitation was repeatedly described as a crucial component of remuneration and was perceived as a burden when having to come out of CHWs pockets, as also a former study conducted in Uganda describes [20]. A previous study conducted in the same rural Ugandan community displays the concerns of patients and HCPs towards CHWs’ capability in managing NCDs [32]. Complementing these previous findings, patients in our study voiced their concerns of current limited CHW knowledge and skills in screening and referring for NCDs. However, participants in our study stated trust and support of a task shifting program if training and supervision structures are in place. In addition, the previous study emphasizes the need of introducing salaries for CHWs’ motivation [32]. HCPs and patients underlined this finding by arguing for monetary compensation of CHWs in task shifting. While we argue, emphasized by our study results, that CHWs need to be formally compensated, facilitated and supervised to perform their roles, the introduction of management structures, supervision and regular trainings throughout CHW programs faces major challenges, as two Ugandan studies acknowledge [2, 19]. Limited funding for Primary Health Care programs in Uganda and uncertainty regarding the cost of CHW programs may inhibit the potentials of continuous facilitation [33]. For the successful introduction of task shifting to CHWs and to address participant concerns, our study emphasizes the need to develop sustainable policy mechanisms that ensure the ongoing training and financial compensation of CHWs. Our findings indicate that these structures will establish the essential community trust.

Secondly, all participant groups presented similar individual characteristics of CHWs that may act as program enablers, while CHWs themselves manifested the findings through personal experiences. Consistent with other findings from Uganda, CHWs presented a high degree of intrinsic commitment and motivation that was nurtured by being recognized in the community and seeing a direct impact of their activities [20, 34]. Confidence of CHWs was identified as a key enabler in providing services, which is in line with discoveries from Mozambique and Indonesia [35, 36]. Social relations with community members or the close-ties to the community were perceived as an enabler but also as a potential barrier by participants. On the one hand, being well known by community members creates a gateway where patients trust CHWs from their own community and feel comfortable in accepting offered services. In contrast, participants described the restrictions and barriers such close ties create. CHWs of a younger age than patients, who are well known within a village since their upbringing, may nurture doubts. Patients often questioned whether younger CHWs are able to fulfill their medical roles. These barriers match a study conducted in Indonesia, where CHWs below an age of 30 were perceived as less trusted and as lacking skills [36]. Hence the closeness to a community may be perceived as a driver but could also be a barrier to CHW-led programs. To mitigate this concern, we recommend undertaking formative research that explores community perceptions prior to program implementation. By taking into account the community’s needs and concerns, CHWs may be stationed in their own home villages or assigned to areas where households have less familiarity with their personal backgrounds.

Thirdly, emotions and behaviors were identified as soft facts, driving or halting a task shifting programs’ success. These findings were mostly derived from patients’ interviews, which portrayed high expectations towards CHWs’ performance and behavior beyond working hours, matching findings from a former Ugandan study [31]. A recent article presents the communities’ expectation of professionalizing the CHW role, training and continuous education [37]. How a CHW is perceived and respected within a community appears as a major indicator of program acceptance. Patients in our study anticipate CHWs to be readily available, empathetic, and competent to serve their health needs while acting as role models in the community. Empathetic health care interventions have proven to create better patient experiences and enhance clinical outcomes [38]. Finally, several patients raised the concern of keeping medical information confidential and therefore distrusting CHWs. Similar results were found in a qualitative study conducted in Western Kenya, where the authors suggest that poor confidentiality may hinder the effectiveness of a chronic disease management program [39].

**Fig. 2:**
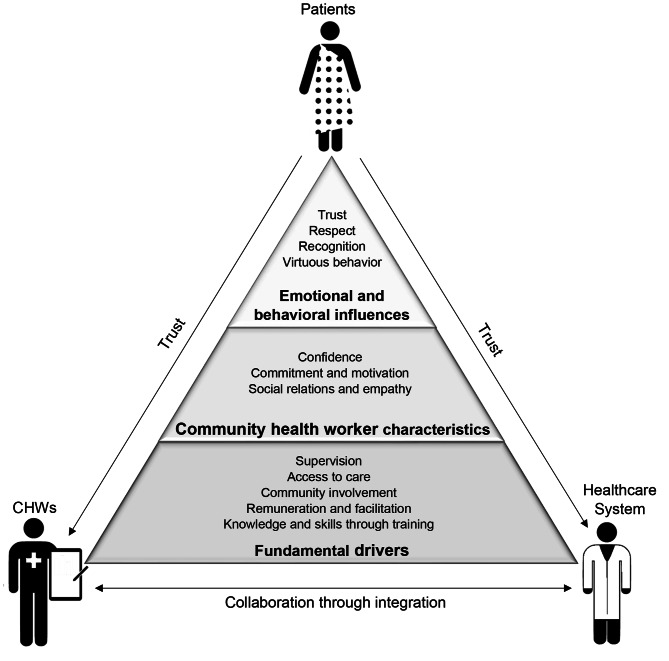
Principles for designing task shifting to community health workers

In Fig. 2 findings are presented in accordance to their level of categorization. It displays the principles when building a CHW-led task shifting program. As outlined above, interventions are built on programmatic, fundamental drivers, further nurtured by CHWs characteristics and finally influenced by socioemotional and behavioral aspects. While the last are strongly dominated by patients’ voices and concerns, CHWs and HCPs appeared less aware of these emotional and behavioral influences that may halt a program’s success. We therefore argue that all stakeholders, namely patients, CHWs, as well as HCPs, need to collaborate and listen to patients’ needs to integrate task shifting successfully within a healthcare system.

In our framework (Fig. 2), we move beyond programmatic components and argue that the establishment of trust-based relationships and communication is a key component of task shifting to CHWs. Trust is built through long-term relationships that do not only focus on household visits carried out by CHWs, but reflect their holistic standing within a community. Several patients reported negative experiences with or observances of CHWs, which may inhibit trust. However, patients appeared open to CHWs services when supervision and training structures are in place and transparently managed. This indicates that these layers are linked and need to be addressed holistically throughout the task shifting program design and implementation. Program implementers and policymakers need to look beyond the structural outlines of an intervention and investigate the social spheres that may challenge community perceptions of trust and the acceptance of such services. A study from South Africa argues that trust can be enhanced through the integration of CHWs in the existing health system such as clinics [40]. As trustful relationships between patients and HCPs were identified in our study, this patient trust may be transferred to CHWs.

This study has a few limitations that are worth noting. Firstly, the research site is unique as it has several years of experience in running community-based screening and education programs. As a qualitative study, this unique experience may make it difficult but not impossible to translate these findings to other rural or urban areas in Uganda or Sub-Saharan Africa. CHWs interviewed presented a unique sample as they were all attached to ACCESS Uganda and previously received training on NCDs. The inclusion of only one medical doctor may have influenced the production of knowledge. As the CHWs are well-known community members, patients’ answers may have been shaped by interpersonal relationships. However, the participants were ensured of confidentiality and encouraged to express their views freely.

The heterogeneous international research team featured “insiders” that share similar attributes with the participants of the study as well as “outsiders” that do not belong to the participating group [41]. Data collection may have been influenced through the research assistants “insider” role as participants may have experienced a heightened sense of ease in the articulation of specific subject matter as a result of common traits and languages with interviewers. Acknowledging research reflexivity, the study outcome of a qualitative study is depending on the researchers’ educational background, profession and even the coding method, which may have concentrated on certain areas of emphasis [42]. Through the collaboration of “insiders” and “outsiders” in the data analysis and interpretation we aimed to achieve a balance in gender, professions and countries of origins. Data analysis and interpretation were driven with the support of “insider” researchers from Uganda; there was no further attempt to validate themes with participants, which poses a limitation of the study.

## Conclusion

CHWs are viewed as a useful resource when task shifting NCD screening and referral for hypertension and diabetes in rural Uganda. The participants in this study – patients, CHWs and HCPs – laid out needs and prerequisites that increase understanding of what drives successful task shifting implementation. In addition, aspects that may serve as barriers for task shifting were identified. The study implies that it is necessary to move beyond the sole hard facts of task shifting to CHWs regarding program design and management. As the established framework presents, building long-term relationships based on trust and collaboration is imperative when establishing community-based task shifting interventions. By addressing fundamental drivers, beneficial perceived CHW characteristics and creating virtuous behavioral influences and positive emotions, CHW programs can flourish and act as a driver against the global health workforce shortage. Further research and policy support is necessary to develop sustainable financing mechanisms for the implementation and scaling-up of such interventions.

## Electronic supplementary material

Below is the link to the electronic supplementary material.

Supplementary Material 1

## Data Availability

The datasets used and/or analyzed during the current study are available from the corresponding author on reasonable request.

## Abbreviations

ACCESS: African Community Center for Social Sustainability
CHW: Community Health Worker
Diabetes: Diabetes Mellitus
FGD: Focus Group Discussion
HCP: Healthcare Professional
HIV: Human Immunodeficiency Virus
IDI: In-depth Interview
LMIC: Low- and Middle-Income Country
NCD: Non-Communicable Disease
VHT: Village Health Teams
WHO: World Health Organization
