# Supplementary material for: Principles for task shifting hypertension and diabetes screening and referral: a qualitative study exploring patient, community health worker and healthcare professional perceptions in rural Uganda
Source: BMC Public Health. 2023 May 12;23:881. doi: 10.1186/s12889-023-15704-w (PMC10176286; doi:10.1186/s12889-023-15704-w)
Supplement: Supplementary file 1 — Supplementary Material 1 [file 12889_2023_15704_MOESM1_ESM.pdf]

Principles for task shifting hypertension and diabetes screening and referral: a qualitative study exploring patient, community health worker and healthcare professional perceptions in rural Uganda

Supplementary Documents

**Table A1. Focus group discussion guide for patients**
1. How does the community perceive CHWs?<br>2. What does the community demand from the CHWs?<br>3. In your views, how are the CHWs in this community selected?<br>*Probes further on:*<br>a. Education level requirements<br>b. What do they do in society?<br>c. Is the community involved?<br>4. What are the major things you expect from the CHWs to do? Or what functions does the CHWs often perform?<br>5. Now were going to talk about NCDs —medical conditions or diseases such as diabetes and hypertension that are non-infectious or non-transmissible in nature. They usually progress slowly, last for long periods of time and are the result of a combination of genetic, physiological, environmental and behaviors factors.<br>a. What are your view of diabetes?<br>b. What are your view of hypertension?<br>6. Concerning your views, do you think the CHWs could be an important human resource when dealing with the screening, identification, and referral for treatment or their prevention?<br>*Probes further on:*<br>a. Tell me about the positive experiences you have had with CHWs.<br>b. Tell me about the negative experiences you have had with CHWs.<br>7. What are your views concerning the CHWs measuring your blood pressure in the community?<br>8. What are your views concerning the CHWs measuring your blood sugar in the community?<br>9. How can CHWs do such a task in the community?<br>*Probes further on:*<br>a. Do you think CHWs can work with other health workers or they alone could do this?<br>b. How can CHWs comfortably do their role?<br>10. Is there anything else you would like to say about CHW screening for diabetes and hypertension in the community? Alternatively, if you were in charge of the CHW program at ACCESS and you were to make changes in the program, what would these changes be?

Principles for task shifting hypertension and diabetes screening and referral: a qualitative study exploring patient, community health worker and healthcare professional perceptions in rural Uganda

Supplementary Documents

**Table A2. In-depth interview guide for community health workers**
1. Can you please tell me about yourself?
2. How did you get recruited as a CHW?
3. What are the roles or things you are meant to do?
Probes further on: How are you facilitated to perform your role in terms of?
a. Training
b. Equipment and Supplies
c. Supervision
d. Incentives
4. Now were going to talk about non-communicable diseases —medical conditions or diseases such as diabetes and hypertension that are non-infectious or non-transmissible in nature. They usually progress slowly, last for long periods of time and are the result of a combination of genetic, physiological, environmental and behaviors factors. How have you been supporting the community in dealing with them?
Probes on: The type of trainings, supplies and tools provided to facilitate the role.
5. Now I want us to talk about motivation of the CHWs, Do you receive any incentives?
Probes on:
a. What are the types of incentives do you receive?
b. How often (regularity and timeliness) do you receive the incentives?
6. Would you be in position to provide support on NCD work?
7. How is your ability to provide diabetes/hypertension screening in the community?
8. What are some of your fears demands to be able to support diabetes and hypertension work?
9. How do you think you can be supported or helped to be able to provide adequate hypertension and diabetes screening and referral?

Principles for task shifting hypertension and diabetes screening and referral: a qualitative study exploring patient, community health worker and healthcare professional perceptions in rural Uganda

Supplementary Documents

**Table A3. In-depth interview guide for healthcare professionals**
1. Could you please tell us about yourself?<br>2. How were the CHWs facilitated or enabled to perform their role in screening and identifying hypertension/diabetes from the community?<br>*Probes further on:*<br>a. How are their roles documented and defined?<br>b. How are the CHWs prepared to perform their roles?<br>c. How are the CHWs equipment?<br>d. How often or how long can they go without supplies?<br>3. Now we are going to talk about NCDs —medical conditions or diseases such as diabetes and hypertension that are non-infectious or non-transmissible in nature. They usually progress slowly, last for long periods of time and are the result of a combination of genetic, physiological, environmental and behaviors factors. Have you been involved with CHWs in their role in diabetes and hypertension care?<br>*Probes further on:*<br>a. How long have you been involved?<br>b. What are their roles of the CHWs?<br>c. How have you facilitated CHWs to perform tasks in hypertension and diabetes?<br>4. Now let us talk about incentives for the VHTs. How are they motivated to perform their roles?<br>5. Is there anything else you would like to say about CHW screening for diabetes and hypertension in the community? Alternatively, if you were in charge of the CHW program at ACCESS and you were to make changes in the program, what would these changes be?
